# HIV-1 Transmission Cluster in Injection Drug Users in Nairobi City, Kenya

**DOI:** 10.4314/ejhs.v33i2.4

**Published:** 2023-03

**Authors:** Sella K Webale, Mark Kilongosi, Godwil Munyekenye, David Onyango, Immaculate Marwa, Nancy Bowen

**Affiliations:** 1 School of Biological sciences, Maseno University, Maseno, Kenya; 2 School of Health Sciences, Kirinyaga University, Kutus, Kenya; 3 National HIV Reference Laboratories, Ministry of Health, Nairobi city, Kenya

**Keywords:** HIV-1, Transmission Cluster, Injection Drug Users, subtype

## Abstract

**Background:**

While there is a striking increase in the prevalence of HIV in injection drug users, information on envelope-gene subtypes and transmission clusters in injection drug users is scarce.

**Method:**

In a cross-sectional study, 247 injection drug users were recruited via out-rich method. Deoxyribonucleic acid was extracted from dry blood spot samples, amplified by Polymerase Chain Reaction and sequenced. Subtyping was performed using COntext-based Modeling for Expeditious Typing (COMET) and Recombinant Identification Program (RIP) tools. Phylogenetic diversity and Transmission clusters were identified using MEGA version 6.0 and TreeLink, respectively.

**Results:**

Overall, 42 (17.0%) injection drug users were sero-positive for HIV-1. Of the 37 samples successfully sequenced, 29 (78.4%) sequences were identified as A1, 6 (16.2%) as AG while 1 (2.7%) as A1/G/AE and A1/C recombinants. The HIV subtypes formed clusters with little genetic diversity.

**Conclusion:**

The high HIV prevalence was associated with transmission clusters and diversity in subtypes indicating ongoing local transmission. Therefore, there is need for comprehensive HIV care tailored to this population.

## Introduction

Two types of HIV are known: the most common HIV-1, which is responsible to the worldwide AIDS epidemic, and the immunologically distinct HIV-2 (1), which is much less common and less virulent but produces clinical findings similar to HIV-1 (1). The HIV-1 type itself includes four groups M (main), O (outlier), N (non-M, non-O), and P (1), which have different geographic distributions but all produce similar clinical symptoms. The M group further splits into 9 subtypes (A1-A4, B, C, D, F1-F2, G, H, J, K), and at least 61 circulating recombinant forms (1). The extensive genetic diversity of HIV-1 is driven by its high replication rate, the error-prone reverse transcriptase, and recombination events that may occur during virus replication (2).

Pathogenicity, clinical progression and transmission of HIV infection vary between *envelope*-gene subtypes (3). Subtype D is associated with rapid progression of disease, relative to subtypes A, C and recombinant viruses (4). By contrast, subtype C is associated with higher HIV-1 RNA load in genital secretion and colostrum and breast milk suggesting increased risk of horizontal and vertical transmission compared to subtypes A and D (5). Immunogens of the HIV-1 *envelope*-gene are prime targets of ongoing vaccine development research (6). Most vaccine formulations currently in clinical trials contain immunogens derived exclusively from subtype B viruses, the predominant genotype in the United States and Europe (7). Despite this clinical, virologic, immunologic and genotypic importance of *envelope*-gene subtypes, there is limited information about HIV-1 *envelope*-gene subtypes in Kenyan injection drug users (8).

The Centers for Disease Control and Prevention (CDC) has recently expanded efforts to use HIV sequence data to identify growing transmission clusters for the purpose of investigation and response (9). HIV transmission cluster may provide a means for identifying segments of the population at highest risk for incident HIV infection (10) offering an opportunity to prioritize clusters of highest public health concern particularly given finite resources for HIV prevention. Kenya is among the countries with the highest HIV burden globally with a national HIV prevalence of 4.9% (11). There is striking increase in HIV prevalence among people who inject drugs in Kenya. A recent survey reported a National HIV prevalence of 18.3% among injection drug users with higher infection rates among IDUs in Nairobi city compared to Mombasa city, suggesting that injection drug use is driving HIV epidemiology in Kenya (12). The intersection of unsafe injecting drug use and unsafe sexual practice is a significant factor for the higher HIV infection rates among injection drug users in Kenya (12). However, information about HIV transmission clusters in Kenyan injection drug users remain scanty (8). As such, the present study assessed if the high HIV infection rates in injection drug users in Nairobi city, Kenya, was associated with viral subtypes and presence of transmission clusters.

## Methods

**Study site and study design**: This was a cross-sectional study targeting HIV infected injection drug users living in Nairobi city, Kenya. Two study sites, Kawangware and Ngara were purposively selected on the basis of existing evidence from previous studies indicating the problem of drug use and especially injecting drug use was rampant in these locations (12). Injection substance users were defined as individuals reporting injecting any illicit drugs from the United Nations Office on Drugs and Crime (UNODC) registry (13), and showing evidence of injection needle-stick scars. HIV infected injection drug users older than eighteen years who signed informed consent were recruited. Injection drug users were recruited by outreach workers, who were mostly former IDUs. The outreach workers (ORWs) were trained to provide information to the injection drug users (IDUs) about the study, which included completing a study questionnaire, sample collection and an HIV rapid test and the opportunity to recruit their friends into the study. Capillary blood was collected on filter paper and used for HIV molecular assays. HIV infected injection drug users older than eighteen years willing to sign informed consent were recruited in this study. Recruited injection drug users were given referral coupons to visit the nearest Drop in Centre (DIC) or health facility for HIV treatment and prevention services.

**HIV molecular assays**: HIV-1 pro-viral DNA was extracted from HIV-1 sero-positive dry blood samples using viral DNA extraction kit according to the manufacturer's protocol (QiaAmp™, Valencia, USA). HIV-1 envelope-gene was amplified by nested polymerase chain reaction (PCR) as described elsewhere (14). PCR products were visualized by ethidium bromide staining of samples electrophoresed on an agarose gel.

Amplified purified PCR products were sequenced directly using Big-Dye Terminator v3.1 Cycle Sequencing kit (Applied Biosystems), on an ABI 3130 xl Genetic Analyzer Program (Applied Biosystems). The resulting forward and reverse HIV-1 *envelope*-gene sequencing chromatograms were scanned for base mis-calls and edited using ChromatoGate (CG) software version 1.2 (15). Pairwise alignment and consensus sequences were generated using DNA Baser Sequence Assembler version 4.20.0 (Heracle Software, Germany). Sequences reported were submitted to GenBank.

**Subtyping HIV-1 envelope-gene**: HIV sequences were subtyped using context-based modelling for expeditious typing (COMET) (16). Sequences identified as “unassigned” by COMET were subsequently analysed by Recombinant Identification Program (RIP) (17) with a window size set at 400 bp and confidence threshold of 90% to determine if they were recombinants. Reference sequences from Los Alamos HIV database were categorized by subtype for RIP analysis.

**Transmission cluster**: Transmission cluster of the HIV-1 sequences of the study participants were identified using TreeLink (18). Phylogenetic inferences by the neighbour-joining method with 1,000 bootstrap replicates under Kimura's two-parameter correction using MEGA 6.06 (19) identified the sequence cluster groups with HIV-1 pure subtypes. Well characterised pure subtype HIV reference sequences from the GenBank were used as background reference.

**Data analysis**: Statistical analysis was performed using Statistical Package for the Social Sciences (SPSS) version 20.0 (IBM SPSS Statistics for Windows, Version 20.0. Armonk, NY: IBM Corp.). Descriptive statistic was used to present information on demographic, clinical and HIV subtypes.

**Ethical considerations**: Ethical approval for this study was obtained from Maseno University Ethical Review Committee. All study participants provided a written informed consent to ensure that autonomy is maintained. There was no monetary reimbursement for recruitment fearing money would further encourage their drug use. Recruited injection drug users were given outreach worker referral coupons to visit the nearest Drop in Centre (DIC) or health facility for HIV testing, treatment and prevention services. Confidentiality was maintained during the study by assigning the participants code numbers.

## Results

**Demographic of study participants**: The demographic and clinical information of the study participants is presented in Table 1. A total of 247 injection drug users were recruited comprising of 223 (90.3%) males and 24 (9.7%) females. Age distribution showed that 57 (23.1%) were between the age of >18≤24 years, 94 (38.1%) were between age >24≤30 while 96 (38.8%) were over 30 years old. Self-reported drug use indicated that 76 (30.8%) abused cocaine, 156 (63.2%) used heroin while 15 (6.0%) used both heroin and cocaine. In addition, 75 (30.4%) abused drugs for a period of less than year, 60 (24.3%) used drug for a period between ≥1≤3 while 112 (45.3%) used the drugs for over 3 years. HIV sero-testing revealed that 42 (17.0%) injection drug users were HIV sero-positive (of whom 12 (28.6%) were on ARV treatment.

**Table 1:** Demographic and clinical information of the study participants

Characteristic	Number (%)
Gender	
Male	223 (90.3)
Female	24 (9.7)
Age	
>18≤24	57 (23.1)
>24≤30	94 (38.1)
>30	96 (38.8)
Drug type injected	
Cocaine	76 (30.8)
Heroin	156 (63.2)
Heroin and cocaine	15 (6.0)
Duration of injection	
<1	75 (30.4)
≥1≤3	60 (24.3)
>3	112 (45.3)
HIV sero-status	
Positive	42 (17.0)
Negative	205 (83.0)
ARV treatment	
No	30 (71.4)
Yes	12 (28.6)

**Figure 1:**
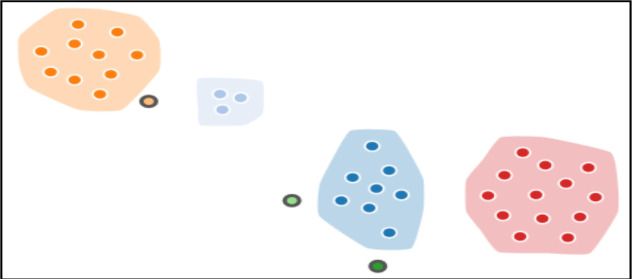
Transmission clusters of HIV-1; a cluster includes all connected individuals, and all are less than 1% divergent from at least one other. Sequences are colored by cluster. Clusters are highlighted and circled. Small circles inside the cluster represent study participant sequences.

**Figure 2:**
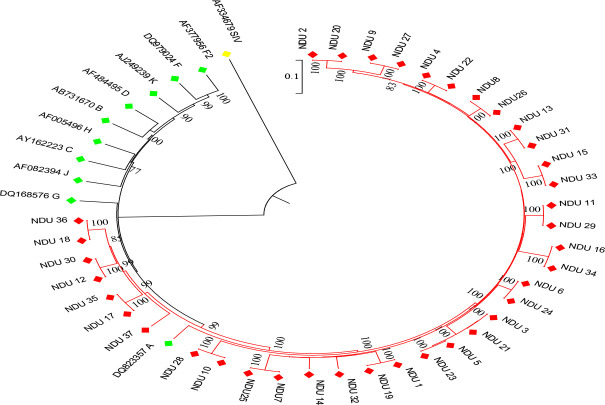
Neighbor-Joining method phylogenetic tree based on 1000 bootsrap replicate. Bootstrap value greater than 70% are shown. Scale bar indicate branch length. Simian Immunodeficiency virus is used as an out-group. Red, yellow and green diamond signs indicate study participants', SIV and HIV reference sequences, respectively. SIV = Simian Immunodeficiency virus reference sequence.

**HIV subtype and transmission clusters**: A total of 37 (88.1%) out of 42 HIV sero-positive samples were successfully sequenced. Sub-typing revealed that 29 (73.0%) isolates were of subtype A1, 6 (16.2%) were AG, while 1 (2.7%) case was reported for A1/C recombinant and A1/G/AE complex recombinants (Table 2). A total of 34 out of 37 sequences clustered with at least one other sequence (Figure 1). Three large clusters, highlighted and circled, each comprising of 13, 10 and 8 sequences stood out. All the 37 (100.0%) sequences clustered within the sequence subtyped as A1 (Figure 2). Nevertheless, the branch lengths separating these sequences on the phylogenetic tree are very short, indicating little genetic diversity.

**Table 2:** HIV subtypes circulating in study participants

Subtype	N (%)
A1	29 (78.4)
AG	6 (16.2)
A1/G/AE	1 (2.7)
A1/C	1 (2.7)

Data are presented as number and proportions (%) of HIV subtypes. A1 = subtype A1; AG = recombinant of subtype A and G; A1/G/AE = complex recombinant of subtype A, G; AE. A1/C = recombinant of subtype A1 and C,

## Discussion

HIV continues to be a major global public health burden having infected over 79 million people and claimed over 36 million lives since the start of the epidemic (20). Ironically, one of the reasons why successful control of HIV is so difficult is the rapid rate of evolutionary change leading to extensive viral diversity both within and between hosts (21). HIV diversity influence virus biological properties, transmission, disease progression, antiviral susceptibility and evolution of antiviral resistance (22). Meanwhile, cluster detection and response help reduce HIV transmission by increasing HIV care and prevention services to people who need (10, 11). Therefore, it is important to determine HIV genetic diversity and transmission clusters in a population.

The present study detected higher prevalence of subtype A1 (78.4%) followed by AG 16.2%), A1/C (2.7%) and A1/G/AE (2.7%) infection among injection drug users. This is partly consistent with previous study reporting higher prevalence of envelope-gene subtype A1 and the remainder being AD, C and complex subtypes in that order, among injection drug users in Nairobi city (8). Studies assessing *in vitro* HIV subtype replication capacity found that A has a higher replication fitness hence increased transmission compared to other subtypes (23, 24), may explain the expansion of subtype A in the present study. It is important to note that, this study detected recombinants of A, an observation similar to previous studies in Kenya (8, 25) suggesting that subtype A is likely to have a high recombination potential. Frequent recombination can occur between subtype A HIV-1 during replication to assort polymorphic sequences and increase genotype diversity of the viral population (26). However, the finding of this study is inconsistent with previous study in Baltimore, Maryland, USA that detected only full-length envelope-gene subtype B in injection drug users (27). HIV subtype distribution vary by geographic region (21)and this may be attributed to genetic variability between ethnicities playing role in determining resistance or susceptibility to HIV-1 subtype infection (24). For example, HLA-B*51:01 allele was protective against HIV-1 subtype B during early infection (28), while being associated with disease progression during HIV-1 subtype C infection (29). Injection drug use may expand HIV subtypes in a country. For example, injection drug users living on boarders play a pivotal role in the cross-border transmission of HIV-1 on borders (30) as well as inside the countries while moving along drug trafficking routes since drug users test heroin purity through self-injection and share needles with traders as part of their drug-purchasing behavior (31). Taken together, there is need for continuous molecular epidemiology surveillance of HIV subtypes circulating in injection drug users to inform vaccine development studies.

Phylogenetic and network analyses based on the full-length *env* gene showed that the sequences from the injection drug users in Nairobi, city, Kenya, more likely clustered with one another indicating increased local HIV transmission networks in this at risk group. This observation is partly consistent with previous studies involving injection drug users in Nairobi, Mombasa and Kisumu cities in Kenya (8) and Romania (32). Even though this was not determined in this study, previous study reported that Kenyan injection drug users transmit HIV through sharing needle and syringes while injecting drugs (12), may explain the transmission clusters observed in this study. HIV-1 infection among this population of injection drug users has another unique characteristic, a very low genetic diversity, a finding that is partly similar to previous study involving injection drug users in Nairobi, Kisumu and Mombasa cities of Kenya (8) and China (31) that reported low interpatient diversity. It is important to note that majority of this population were not on antiretroviral therapy which has been shown to drive intra-host HIV genetic diversity (33). Meanwhile, much higher inter-patient genetic distance has been reported among female sex workers and their elderly male clients in China (34). The disparity in genetic distances may be due to transmission bottlenecks whereby mucosal membrane selects for minor variants during sexual transmission, while virus circulating among IDUs has direct transmission into the bloodstream, and the most abundant form may repeatedly establish itself (35). Therefore, there is need for harm reduction strategies to reduce HIV transmission in this at risk population.

This study has limitations. We acknowledge the relatively small sample size of HIV infected study participants in our study. Subtypes were based on the *envelope*-gene region in all the study participants. Even though ARV drug testing can help identify persons who are on ART (36), HIV treatment status in this study was based on self-report.

In conclusion, the striking increase in prevalence of HIV in injection drug users is associated with transmission clusters and pure and recombinant subtypes suggesting that there has been an active injection drug using network between IDUs in Nairobi city, Kenya. It is necessary to implement more effective and comprehensive HIV prevention and control services for further control of HIV-1 dissemination in this population.
